# Cytotoxicity of *Aspergillus* Section *Fumigati* Isolates Recovered from Protection Devices Used on Waste Sorting Industry

**DOI:** 10.3390/toxins14020070

**Published:** 2022-01-20

**Authors:** Carla Viegas, Magdalena Twarużek, Marta Dias, Elisabete Carolino, Ewelina Soszczyńska, Liliana Aranha Caetano

**Affiliations:** 1 H&TRC—Health & Technology Research Center, ESTeSL—Escola Superior de Tecnologia da Saúde, Instituto Politécnico de Lisboa, 1990-096 Lisbon, Portugal; martasfd@gmail.com (M.D.); etcarolino@estesl.ipl.pt (E.C.); liliana.caetano@estesl.ipl.pt (L.A.C.); 2 NOVA National School of Public Health, Public Health Research Centre, Universidade NOVA de Lisboa, 1099-085 Lisbon, Portugal; 3 Comprehensive Health Research Center (CHRC), NOVA Medical School, Universidade NOVA de Lisboa, 1169-056 Lisbon, Portugal; 4 Department of Physiology and Toxicology, Faculty of Biological Sciences, Kazimierz Wielki University, Chodkiewicza 30, 85-064 Bydgoszcz, Poland; twarmag@ukw.edu.pl (M.T.); eweso@ukw.edu.pl (E.S.); 5 Research Institute for Medicines (iMed.ULisboa), Faculty of Pharmacy, University of Lisbon, 1649-003 Lisbon, Portugal

**Keywords:** cytotoxicity, protection devices, MTT assay, risk assessment, waste sorting environment

## Abstract

Safe working conditions must be guaranteed during waste sorting, which is crucial to maximizing recycling and reuse, in order to minimize workers’ exposure to chemical and biological hazards. This study determines the contribution of *Aspergillus* section *Fumigati* to the overall cytotoxicity of filtering respiratory protection devices (FRPD) and mechanic protection gloves (MPG) collected in 2019 from different workstations in one waste sorting industry in Portugal. The cytotoxicity of 133 *Aspergillus* section *Fumigati* isolates was determined as IC50 in human A549 epithelial lung cells and swine kidney cells, using the MTT (3-(4,5-dimethylthiazol-2-yl)-2,5-diphenyltetrazolium bromide) assay. *Aspergillus* section *Fumigati* cytotoxicity results were compared with previous total cytotoxicity data from FRPD and MPG samples. A significant correlation was detected between the total cytotoxicity of samples and cytotoxicity of *Aspergillus* section *Fumigati* isolates in A549 cells (rS = −0.339, *p* = 0.030). The cytotoxicity of *Aspergillus* section *Fumigati* isolates explained 10.7% of the total cytotoxicity of the sample. On the basis of the comparison of cytotoxicity levels, it was possible to determine the contribution of *Aspergillus* section *Fumigati* isolates for the total cytotoxicity of protection devices used in the waste sorting industry. The results support in vitro toxicology as a relevant approach in risk assessments regarding cytotoxicity in passive sampling, and thus, useful in determining the contribution of relevant microbial contaminants to overall cytotoxicity. This approach can provide valuable answers in dose/response studies, and support innovations in risk characterization and their translation into occupational policies.

## 1. Introduction

European countries have established a reduction in the amount of waste produced to ensure full implementation of the waste policy targets in all member states [1]. Additionally, a circular economy increases waste recovery and thus, leads to a great workforce engaged in waste management. Waste sorting industries are one of the key points to achieving the European Union milestones and Sustainable Development Goals (SDGs) proposed by the World Health Organization. It is crucial to raise waste sorting to maximize recycling and reuse and, in parallel, to ensure safe working conditions. Therefore, although improved waste management contributes to reducing environmental negative impacts and health outcomes, the exposure of workers in waste sorting to microorganisms may increase, leading to a negative impact on workers’ health.

It has been reported that workers in waste sorting plants (WSP) are exposed to a complex mixture of contaminants of chemical and biological origin [2,3,4,5]. These mixtures of contaminants, difficult to entirely characterize, are most certainly responsible for biological responses that can result in health disorders [6,7,8]. Despite information on health outcomes obtained from epidemiological studies, these are not enough to establish a link between exposure and health problems, leading to the unavailability of occupational exposure limit values (OELs) for airborne biological agents [9]. The health outcomes caused by exposure to bioaerosols are linked to the specific microorganisms to which workers are exposed. Therefore, it is extremely important to characterize very well the toxigenic potential of each microorganism. 

*Aspergillus* section *Fumigati* (one section of the *Aspergillus* genus) was reported as one of the most prevalent fungal species in filtering respiratory protection devices (FRPD) and mechanic protection gloves (MPG) [10,11], as in other environmental samples [4,7], from the same waste sorting unit. Moreso, *the Fumigati* section was the most abundant *Aspergillus* section detected on the interior layers (IL) (33.33%, 40 samples out of 120) and on the exhalation valves (EV) (1.66%, 2 samples out of 120) of FRPD [10]. Regarding MPG, the *Fumigati* section was the second most prevalent among *the Aspergillus* genus on DG18 (23.17%) [11].

*Aspergillus* section *Fumigati* is the *Aspergillus* section more often associated with respiratory symptoms due to the small size of the conidia and other virulence factors [12,13]. Of note, the development of resistance to antifungal drugs among *Aspergillus* sections, mainly in section *Fumigati*, is a phenomenon with growing prevalence in Europe that has been related to therapeutic failure and high mortality rates, mostly due to opportunistic invasive fungal infections among immunocompromised individuals [14].

Although it is generally accepted that fungal exposure is unhealthy, and measures must be taken to limit fungi development, the available literature on adverse health effects from exposure to airborne toxigenic fungi is scarce or lacks clarity in establishing the link between exposure and the observed biological effects. Long-term exposure to toxigenic fungi has been suggested as being disruptive of natural killer cell activity, causing symptoms such as headaches, fever, cough, depression, anxiety, among others [15]. Other studies reported exposure to fungi and bioaerosols as inducers of immunosuppression and inflammation [16,17]. 

Without weakening the features of molecular analysis, culture-based methods are still critical to assess the viability of pathogenic microorganisms related to their infectivity potential. Furthermore, a microorganism’s viability is associated with the potential of inflammatory and cytotoxic responses and, consequently, the infection potential. Therefore, molecular tools must be used in parallel with classic methods [18,19,20]. In addition, in exposure assessment studies, where the exposure is mainly occurring by inhalation, the outcomes of respiratory diseases may vary significantly with fungal viability, and thus, it is of utmost importance to apply cultures for the fungal assessment or isolates recovery [21]. This corroborates the importance to perform the identification by culture, using the same procedure as in clinical samples, since it is the gold standard for the diagnosis of fungal infections and allows susceptibility testing [22,23].

In the current study, the contribution of *Fumigati* isolates from the *Aspergillus* section to the cytotoxicity of FRPD and MPG samples from the waste sorting industry was determined. The research is part of a wider project consisting of a multi-faceted pilot study aiming to analyze the cytotoxicity of protection devices used in the waste sorting industry. In this study, the cytotoxicity of *Fumigati* isolates was determined as IC50 in two different mammalian cell lines using the 3-(4,5-dimethylthiazol-2-yl)-2,5-diphenyltetrazolium bromide (MTT) assay and were compared to previous results obtained with composite FRPD and MPG samples. In addition, a detailed statistical analysis was conducted with the aim of evaluating the relations between the cytotoxic effect of *Fumigati* isolates and the total cytotoxicity of the FRPD and MPG samples.

## 2. Materials and Methods

### 2.1. Protection Devices’ Sampling and Sample Preparation

The sampling of protection devices was the same as Viegas et al. 2020 [10,11]. Briefly, over 180 protection devices used by waste workers (namely, FRPD (N = 120) and MPG (N = 67)) were randomly collected on a weekday during the winter season (between January and February 2019) in four workstations (Table S1—Supplementary Material) at one waste sorting industry located in Lisbon, Portugal. Samples were kept refrigerated and processed in aseptic conditions, as previously reported [10,11], and characterized regarding their microbial contamination and cytotoxic effect [10,11,24]. 

### 2.2. Microbiological Characterization and Cytotoxicity Evaluation of FRPD e MPG

Selective culture media for fungi were used to allow fungal growth (malt extract agar (MEA) supplemented with chloramphenicol (0.05%); dichloran-glycerol agar (DG18)) and to screen azole resistance (Sabouraud dextrose agar (SDA) supplemented with 4 mg/L itraconazole (ITR), 1 mg/L voriconazole (VOR), 0.5 mg/L posaconazole (POS), or non-supplemented (adapted from EUCAST 2018)). FRPD and MPG, without prior use, were used as negative controls. All samples were characterized regarding fungal contamination (fungi colony-forming units, CFU.m^−2^) and diversity (morphological characterization and molecular detection) [10,11]. Microscopic mounts were performed using a tease mount or Scotch tape mount and lactophenol cotton blue mount procedures, and the morphological identification from all of the fungi was performed using macro- and microscopic characteristics, as reported before [10,11].

### 2.3. Collection of Aspergillus Section Fumigati Isolates

**Figure 1:**
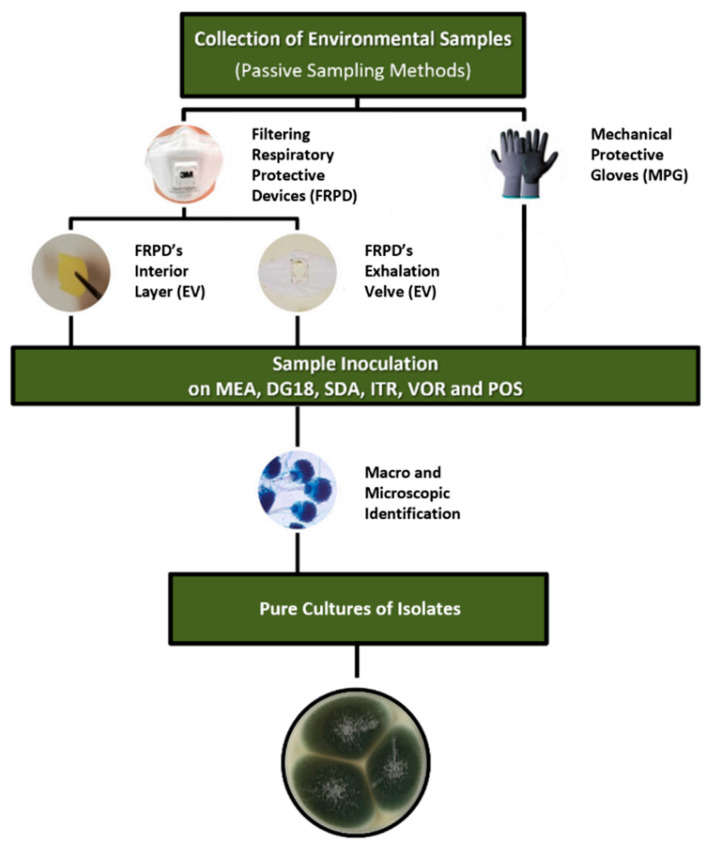
Protocol applied for the *Aspergillus* section *Fumigati* isolates collection.

*Aspergillus* section *Fumigati* isolates were extracted from each FRPD-IL, EV and MPG sample after its identification in at least one media (MEA, DG18, SDA, or azole-supplemented SDA). Only one isolate from one media per sample was used. We selected the one with a higher possibility to obtain a pure culture of the Fumigati isolate without contamination, following the procedures already published [25] (Figure 1).

A total of 133 isolates from *Aspergillus* section *Fumigati* were recovered. Considering each matrix, the one with the highest prevalence of isolates was the IL from the FRPD (FRPD-IL) with a total of 61 isolates, followed by the 50 isolates from EV (FRPD-EV). 

The media with the highest number of isolates was MEA, with a total of 53 isolates, followed by SAB with a total of 48 isolates. It is important to highlight that there were 4 isolates recovered (2 from FRPD-IL; 2 from FRPD-EV) growing on ITR, and 2 isolates from FRPD-IL growing on VOR. No isolates were recovered from POS (Table 1).

### 2.4. Cell Lines and Culture Conditions

Two mammalian cell lines were used to screen the cytotoxicity of the *Aspergillus* section *Fumigati* isolates. Swine kidney (SK) cells, derived from a cell culture collection of the Ludwig-Maximilians-Universität München, were kindly provided by Prof. Manfred Gareis, Ph.D., in 2011. Human A549 epithelial lung cells were purchased from the ATCC collection. The cells were maintained in Eagle’s Minimum Essential Medium (MEM) supplemented with 10,000 units of penicillin and 10 mg of streptomycin per mL in 0.9% NaCl (Sigma-Aldrich, St. Louis, MO, USA) and fetal bovine serum (Sigma-Aldrich, St. Louis, MO, USA). 

### 2.5. Cytotoxicity Evaluation

Extracts containing an equivalent of *Aspergillus* section *Fumigati* grown on one 62.5 cm^2^ surface Petri dish were prepared as follows: colonies of the fungi were grown on Czapek Dox agar at 25 °C for 2 weeks, and the following 2 weeks in 10 °C. Then, colonies of *Aspergillus* section *Fumigati* were removed from Petri dishes with a sterile scalpel and transferred to a sterile bag (BagLight^®^, Interscience, Dubai, Emirates United States). Then, 50 mL of chloroform was added and homogenized for 5 min in a laboratory blender (BagMixer 400, Interscience). The mycelium was harvested by filtration through Whatman filter paper. The samples were evaporated to dryness. The received extracts were dissolved in 1 mL of mixture of ethanol dimethyl sulfoxide-minimum essential medium with Earle’s salts (MEM) (1.7 + 0.3 + 98, *v*/*v*/*v*. Then, serial log 2 dilutions of the sample extract were prepared. 

The A549 and SK cells were used to evaluate the cytotoxic effect of *Aspergillus* section *Fumigati* isolates. The cell cultures were harvested using 0.25% (*w*/*v*) Trypsin 0.53 mM EDTA, suspended in the culture medium. After cell count using the Scepter ™ 2.0 Cell Counter (Merck), A549 and SK cells were transferred (100 µL) to a 96-well plate (densities of 2.5 × 10^5^ cells/mL) and exposed to serial log 2 dilutions of sample extracts for 48 h at 5% CO_2_, 37 °C, and humid atmosphere. The cytotoxicity level was measured by reduction of MTT tetrazolium salt to formazan at 510 nm [26] (ELISA LEDETECT 96, BioMed Dr. Wieser GmbH; MikroWin 2013SC software). The lowest concentration of sample extracts causing a drop in absorption to <50% of cell division activity (IC50) was considered the threshold toxicity level.

### 2.6. Statistical Analysis

The data were analyzed using the statistical software SPSS V26.0 for windows. The results were considered significant at the 5% significance level. To test the normality of the data, the Shapiro–Wilk test was used. For the comparison of the IC50 values both in the sample and in the *Aspergillus* section *Fumigati* isolates between the sample type (FRPD-IL and EV; MPG) and between the media, the Kruskal–Wallis test was used since the assumption of normality was not verified. To study the relationship between the IC50 values of A549 and SK cells between the sample and the *Aspergillus* section *Fumigati* isolates, Spearman’s correlation coefficient was used. To evaluate the influence of the values of the *Aspergillus* section *Fumigati* isolates on the sample values, simple linear regression analysis was used, with the model checking the Gauss–Markov conditions.

## 3. Results

### 3.1. Aspergillus Section Fumigati Cytotoxicity

**Figure 2:**
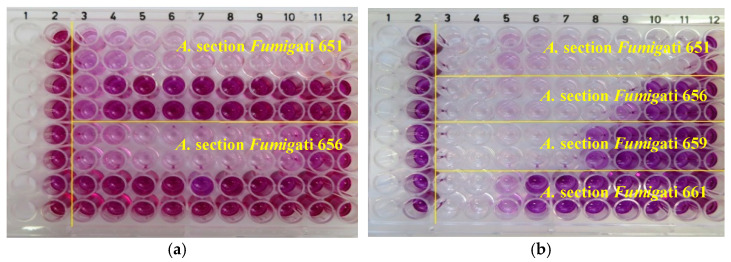
Photographs of the MTT micro-plates with extracts containing the *Aspergillus* section *Fumigati* recovered from SAB media (651, 656, and 659) and from ITR medium (661): (**a**) A549 cells; (**b**) SK cells.

*Aspergillus* section *Fumigati* was screened for cytotoxicity in A549 and SK cells, in order to determine their contribution to the overall cytotoxic effect previously observed in FRPD (IL and EV) and MPG samples (Supplementary Material—Tables S1–S4). The obtained results are presented in Table 2. An example of *Aspergillus* section *Fumigati* sample extracts’ cytotoxicity is presented in Figure 2a,b.

The source of fungi (FRPD-IL, FRPD-EV or MPG) had no statistically significant differences for IC50 in A549 cells (χ^2^ (2) = 0.906, *p* = 0.636) or SK cells (χ^2^ (2) = 2.979, *p* = 0.225). A statistically significant lower cytotoxic effect was detected for *Aspergillus* section *Fumigati* grown on ITR media in SK cells (χ^2^ (2) = 8058, *p* = 0.045). 

### 3.2. Correlation Analysis

In A549 cells, there was a significant and inverse correlation between whole sample IC50 values and *Aspergillus* section *Fumigati* isolates IC50 values (rS = −0.339, *p* = 0.030), indicating that greater IC50 of *Aspergillus* section *Fumigati* isolates was associated with lower IC50 of the whole sample (Table 3). These findings reveal that *Aspergillus* section *Fumigati* isolates account for just 10.7% of the cytotoxicity found in the whole sample from which it was recovered, implying that other components of the sample account for the remaining 89.3%.

## 4. Discussion

Bioassays to assess the toxicity of microorganisms present at workplaces enable the identification of risk factors for workers’ health. Current trends in European policies dedicated to waste management, fostered by the implementation of the Sustainable Development Goals worldwide, reinforce this endeavor as of critical importance, due to the increase in the workforce engaged in waste sorting. The characterization of the toxigenic potential of microorganisms present in the waste sorting workplaces is, however, a major challenge. This assessment includes air sampling and passive sampling, and might include individual protection devices [11,24]. 

The choice of appropriate cell lines is critical when conducting bioassays to elucidate induced toxicity in humans. The human A549 epithelial lung cell line is largely used in lung cell biology [27] and was applied in this study as a model for alveolar cells, while the swine kidney (SK) cells are a valid alternative to primary human cells for renal in vitro toxicology, due to high similarity in renal physiology [28]. The 3-[4,5, dimethylthiazol-2-yl]-2-5 diphenyltetrazolium (MTT) tetrazolium salt assay has commonly been used to measure cytotoxicity in different cell lines, comprising cell lines of animal and human origin [7,29] and indicates cell respiration competence and metabolic activity. Prinsloo and colleagues have already used the MTT assay to determine the effect of microbial contaminants on cell viability [30].

Based on biological responses obtained from the mycobiota assessment in the environment, such as cytotoxicity or inflammasome, conclusions about potential health risks following specific exposure routes can be drawn. Aside from more common mycotoxins’ assessments, other fungal substances, such as cytoplasmatic and cell wall components, can be released into the environment by fungal hyphae and exert noxious health effects [31,32]. Fungal cytotoxicity, determined by in vitro evaluation of fungal contaminated building materials, has been reported to be linked to the quality and quantity of mycotoxigenic fungi, rather than with mycotoxins alone [33]. The cytotoxicity of fungal extracts is further confirmed by other studies, which concluded that fungal extracts exerted a cytotoxic effect and presented a potential health risk following exposure by inhalation of fungal spores of common indoor molds [34]. Of note, while the exposure to airborne fungal might predispose to fungal colonization of the airways without infection [35], it is a relevant risk factor for infection in more sensitive individuals, such as workers suffering from asthma. 

Recent studies from our group assessing the cytotoxic effect of contamination present in MPG and in FRPD used in the waste sorting, also revealed a cytotoxic effect of both in mammalian cell lines, being higher in MPG than in FRPD in SK cells [36]. This might be related to the time of use. Moreso, MPG were used in more than one work shift, while FRPD were replaced in each work shift, thus, justifying the higher cytotoxicity observed in SK cells. Indeed, the number of hours of use was found to be positively correlated with microbial contamination found on MPG and SK cells [11]. 

In the present study, the *Aspergillus* section *Fumigati* recovered from the whole FRPD and MPD samples were screened for cytotoxicity in A549 and SK cells using the MTT assay to elucidate the contribution of this fungal species for overall cytotoxicity. It was observed that the cytotoxicity of *Aspergillus* section *Fumigati* contributed only 10.7% to the cytotoxicity of the whole sample. The cytotoxicity of the whole sample might be related to a wide array of contaminants and pollutants that are common in the waste sorting industry [37]. Aside from organic contaminants, inorganic components (not analyzed in this study), such as metals (lead and chromium), persistent organic pollutants (polycyclic aromatic hydrocarbons (PAHs)), bisphenols, phthalates, and brominated flame retardants that originate from the residues sorted in the waste sorting unit, might also be present and have a cytotoxic effect, particularly in lung cells [38]. Moreso, a critical aspect of the waste sorting occupational environment is that workers are exposed to a complex mixture of chemical and biological contaminants which complete characterization, though urgent, is difficult to achieve with standard sampling methods and reproducible data [7,8]. This aspect may explain the contribution of only 10.7% of the *Fumigati* section to the general cytotoxicity of the samples. However, we should consider that cytotoxicity effects found on FRPD and MPG are most likely linked to microbial contamination than chemical pollutants since high levels of chemical contaminants would cause acute toxicity among workers, whereas low chemical concentrations rarely have an effect on cell viability [19,30].

A cytotoxicity evaluation of fungi belonging to genus *Aspergillus* collected from various hospital wards [39] concluded that *Aspergillus* species were cytotoxic in 79% of the cases, with significant differences in the average cytotoxicity, depending on the species (*A. ochraceus*, *A. flavus* and *A. niger* species), although no conclusions could be drawn on which species was more cytotoxic. These results were much higher than the previously obtained with fungal spores from humid domestic environments, in which 47% of the evaluated fungi displayed cytotoxicity in vivo [40]. Regarding cytotoxicity depending on fungal species/sections, *Aspergillus* section *Fumigati* has been reported to be more cytotoxic than other *Aspergillus* sections [41,42]. Some bioactive fungal compounds from *Aspergillus* species have been described as presenting cytotoxicity, namely, bisabolone sesquiterpenoid derivatives isolated from *Aspergillus tennesseensis* [43]. Some of these compounds were described to present cytotoxicity against A549 cells with IC50 ranging from 44 to 61 μM [43]. In future studies, to compare the cytotoxicity between different *Fumigati* isolates, genetic characterization from isolates will be ensured [25,44]. 

Additional assays or the use of additional cell lines and other biomarkers will be important to better estimate the cytotoxicity of *Aspergillus* section *Fumigati* isolates. Furthermore, in vitro toxicology should always be combined with an accurate assessment of exposure to microbial and chemical contamination. This should be the followed approach to achieve useful information on the potential health effects of co-exposure to multiple stressors [24].

In order to fill the data gaps of concern for regulatory authorities (risk assessors and/or risk managers), toxicological studies need to be developed for the assessment (in vitro) of mixtures relevant to occupational health. Due to the lack of information on all the risk factors that are present in waste sorting plants [2,3,4,5], that are deeply dependent on the kind of waste that is being sorted, and the difficulty to accurately assess the exposure to all the pollutants, it is critical to perform the overall cytotoxicity from the sample extracts as a cytotoxicity pre-screening, as was the case of the previous studies performed to FRPD and MPG [11,24]. After identifying the potential indicators of each kind of pollutants—*Aspergillus* section *Fumigati* for mycological contamination—the assessment of their own contribution to the overall cytotoxicity might help to unveil potential health risks of exposure. 

## 5. Conclusions

This study made it possible to determine the contribution of isolates from the *Aspergillus* section *Fumigati* to the total cytotoxicity of the protection devices analyzed. These results support in vitro toxicology as a suitable approach to follow in risk assessments in settings with a high burden of microbial contamination, such as waste sorting occupational environment as follows: first, screening of the overall cytotoxicity of passive samples; and second, to assess the contribution of relevant indicators for each group of pollutants (microbial, chemical) to general cytotoxicity. This approach of focusing on in vitro toxicology can provide valuable answers in dose/response studies, and support innovations in risk characterization and their translation into occupational policies.

## Figures and Tables

**Table 1:** The number of *Aspergillus* section *Fumigati* isolates recovered from different sources per culture media.

Source	MEA	DG18	SAB	ITR	VOR	POS	Total
FRPD-IL	24	11	22	2	2	0	61
FRPD-EV	21	14	13	2	0	0	50
MPG	8	1	13	0	0	0	22
TOTAL	53	26	48	4	2	0	133

**Table 2:** Frequencies of threshold toxicity level (IC50) of *Aspergillus* section *Fumigati*.

Dilution Step	IC50	A549	SK
		*N*	*N*
1	31.250 cm^2^/mL	1	2
2	15.625 cm^2^/mL	1	2
3	7.813 cm^2^/mL	4	3
4	3.906 cm^2^/mL	1	3
5	1.953 cm^2^/mL	0	3
6	0.977 cm^2^/mL	2	6
7	0.488 cm^2^/mL	3	11
8	0.244 cm^2^/mL	4	20
9	0.122 cm^2^/mL	27	31
10	0.061 cm^2^/mL	48	52
11	3.050 mm^2^/mL	25	0
12	1.525 mm^2^/mL	10	0
13	0.7625 mm^2^/mL	3	0
14	0.3812 mm^2^/mL	2	0
15	0.1906 mm^2^/mL	1	0
16	0.0953 mm^2^/mL	1	0

**Table 3:** Study of the relationship between IC50 A549 and SK cells between the sample and the *Aspergillus* section *Fumigati* isolates. Spearman correlation results.

		IC50 in the Sample	IC50 *Aspergillus* Section *Fumigati* Isolates	
		SK	A549	SK
IC50 in the sample	A549	0.667	−0.339 *	−0.157
	SK		0.247	−0.128
IC50 *Aspergillus* section *Fumigati* isolates	A549			0.076

* Correlation is significant at the 0.05 level (2-tailed).

## Supplementary Materials

The following are available online at https://www.mdpi.com/article/10.3390/toxins14020070/s1, Table S1: IC50 values (mm^2^/mL) of FRPD, per workstation, determined by the dilution method in A549 and SK cells (MTT assay); Table S2: IC50 values (mm^2^/mL) of gloves, determined by the dilution method in HepG2 and SK cells (MTT assay); Table S3: Heatmap for IC50 values (mm^2^/mL) in the sample from FRPD and MPG, determined by the dilution method in A549 and SK cells (MTT assay); Table S4: Heatmap for IC50 values (mm2/mL) in the sample from FRPD and MPG, determined by the dilution method in A549 and SK cells (MTT assay).
