# Supplementary material for: Cytotoxicity of Aspergillus Section Fumigati Isolates Recovered from Protection Devices Used on Waste Sorting Industry
Source: Toxins (Basel). 2022 Jan 20;14(2):70. doi: 10.3390/toxins14020070 (PMC8879639; doi:10.3390/toxins14020070)
Supplement: Supplementary file 1 [file toxins-14-00070-s001.zip › toxins-1484131-supplementary.pdf]

Supplementary Materials: Cytotoxicity of *Aspergillus* Section *Fumigati* Isolates Recovered from Protection Devices Used on Waste Sorting Industry

Carla Viegas, Magdalena Twarużek, Marta Dias, Elisabete Carolino, Ewelina Soszczyńska and Liliana Aranha Caetano

Table S1. The number of FRPD and MPG collected in each workplace. Workstations were identified as the ones where workers would spend more time wearing these protection devices, and with identified increased risk of exposure to microbiologic agents [10,36].

Workstations	Tasks	FRPD Number	MPG Number
FMW	Feeding machines with waste	33	9
SW	Sorting waste	54	40
MI	Machines inspection	12	10
MSVO	Machines and special vehicles operator	13	8
Not specified (without information)	--------------	8	--------------
**Total**		**120**	**67**

2.1. Protection Devices' Sampling and Sample Preparation

1. FRPD and MPG cytotoxicity

Previous studies assessed the cytotoxicity of the FRPD interior layer collected at different workstations [36] and compared it to FRPD exhalation valves [19] using two in vitro models: A549 and SK cells. A549 cells were used as an in vitro model for exposure through inhalation, and SK cells as a mammalian cell model for renal toxicity, mainly described for mycotoxins. A third study assessed the cytotoxicity of MPG in SK and HepG2 cells [11]. A different cell line was used in this study, namely, HepG2 cells, as a model for hepatic toxicity, besides SK cells, to assess renal toxicity. These results are summarized here in order to further understand the contribution of Aspergillus sp. to the observed cytotoxicity.

2.1.1. Cytotoxicity of FRPDs' interior layers and exhalation valves

As previously reported, IC50 values of FRPD-IL ranged from 1.25 to 5 mm2/mL in A549 and 5 mm2/mL in SK cells [36]. With FRPD-EV, IC50 values ranged from 5 to 10 mm2/mL in both cell lines [19] (Table S2).

Table S2. IC50 values (mm^2/mL) of FRPD, per workstation, determined by the dilution method in A549 and SK cells (MTT assay).

Workstation	**IC_{50} (mm^2/mL)**															
	**Interior Layer [1]**								**Exhalation Valves [2]**							
	**A549**				**SK**				A549				SK			
	**10**	**5**	**2.5**	**1.25**	**10**	**5**	**2.5**	**1.25**	10	**5**	**2.5**	**1.25**	10	**5**	**2.5**	**1.25**
FMW (n=40)	0	**23**	0	0	0	2	0	0	**13**	0	0	0	5	0	0	0
MI (n=4)	0	2	0	0	0	0	0	0	0	0	0	0	0	0	0	0
MSVO (n=13)	0	5	1	0	0	0	0	0	3	0	0	0	0	0	0	0
SW (n=53)	0	**23**	2	1	0	2	0	0	**18**	1	0	0	1	1	0	0
n.s. (n=8)	0	5	0	0	0	1	0	0	4	0	0	0	0	0	0	0
Total	*0*	*58*	*3*	*1*	*0*	*5*	*0*	*0*	*38*	*1*	*0*	*0*	*6*	*1*	*0*	*0*

[1] Results from Viegas et al. (2020d); [2] Results from Viegas et al. (2021).

2.1.2. Cytotoxicity of MPGs

In the study conducted to assess the cytotoxicity of MPG [11], IC50 values ranged from 0.02 to 5 mm2/mL in HepG2 cells and from 0.04 to 5 mm2/mL in SK cells (Table S3).

Table S3. IC50 values (mm^2/mL) of gloves, determined by the dilution method in HepG2 and SK cells (MTT assay).

	Samples (N)	
**IC_{50} (mm^2/mL)**	**Hep G2**	**SK**
**5**	8	**21**
**2.5**	3	**14**
**1.25**	5	5
0.625	**13**	6
0.3125	5	6
0.25	1	0
0.156	3	3
0.08	1	0
0.04	**17**	1
0.02	1	0
n.d.	10	13

With regard to the A549 cells' results from the extracts' samples (FRPD), statistically significant differences were detected ($U = 18.00$, $p < 0.0001$), with significantly higher values in the ones recovered from EV, which reveals less cytotoxicity (Table S4).

Regarding SK cells in the extract samples, statistically significant differences were also detected between the sample ($\chi 2(2) = 8.838$, $p = 0.012$), with significantly higher values in the EV (less cytotoxicity) and significantly lower values in the MPG or more cytotoxicity (Table S4).

Table S4. Heatmap for IC50 values (mm^2/mL) in the sample from FRPD and MPG, determined by the dilution method in A549 and SK cells (MTT assay).

			IC50 in the Sample		
			**HepG2**	**A549**	**SK**
			**Median**	**Median**	**Median**
**Sample type**	**FRPD**	IL		5.00	5.00
		EV		10.00	10.00
	MPG		0.438		2.50
